# Genomic Diversity, Antimicrobial Resistance, Plasmidome, and Virulence Profiles of *Salmonella* Isolated from Small Specialty Crop Farms Revealed by Whole-Genome Sequencing

**DOI:** 10.3390/antibiotics12111637

**Published:** 2023-11-18

**Authors:** Menuka Bhandari, Jelmer W. Poelstra, Michael Kauffman, Binta Varghese, Yosra A. Helmy, Joy Scaria, Gireesh Rajashekara

**Affiliations:** 1 Center for Food Animal Health, Department of Animal Sciences, College of Food, Agricultural, and Environmental Sciences, The Ohio State University, Wooster, OH 44691, USA; bhandari.72@osu.edu (M.B.); kauffman.42@osu.edu (M.K.); 2 Molecular and Cellular Imaging Center, College of Food, Agricultural, and Environmental Sciences, The Ohio State University, Wooster, OH 44691, USA; poelstra.1@osu.edu; 3 Department of Veterinary Pathobiology, Oklahoma State University, Stillwater, OK 74074, USA; binta.varghese@okstate.edu (B.V.); joy.scaria@okstate.edu (J.S.); 4 Department of Veterinary Science, Martin-Gatton College of Agriculture, Food and Environment, University of Kentucky, Lexington, KY 40546, USA; yosra.helmy@uky.edu

**Keywords:** *Salmonella*, whole-genome sequencing, serotype, antimicrobial resistance genes, virulence genes, small specialty crop farms

## Abstract

*Salmonella* is the leading cause of death associated with foodborne illnesses in the USA. Difficulty in treating human salmonellosis is attributed to the development of antimicrobial resistance and the pathogenicity of *Salmonella* strains. Therefore, it is important to study the genetic landscape of *Salmonella*, such as the diversity, plasmids, and presence antimicrobial resistance genes (AMRs) and virulence genes. To this end, we isolated *Salmonella* from environmental samples from small specialty crop farms (SSCFs) in Northeast Ohio from 2016 to 2021; 80 *Salmonella* isolates from 29 *Salmonella*-positive samples were subjected to whole-genome sequencing (WGS). In silico serotyping revealed the presence of 15 serotypes. AMR genes were detected in 15% of the samples, with 75% exhibiting phenotypic and genotypic multidrug resistance (MDR). Plasmid analysis demonstrated the presence of nine different types of plasmids, and 75% of AMR genes were located on plasmids. Interestingly, five *Salmonella* Newport isolates and one *Salmonella* Dublin isolate carried the ACSSuT gene cassette on a plasmid, which confers resistance to ampicillin, chloramphenicol, streptomycin, sulfonamide, and tetracycline. Overall, our results show that SSCFs are a potential reservoir of *Salmonella* with MDR genes. Thus, regular monitoring is needed to prevent the transmission of MDR *Salmonella* from SSCFs to humans.

## 1. Introduction

*Salmonella* is a rod-shaped, facultative anaerobic, Gram-negative bacterium in the Enterobacteriaceae family that causes salmonellosis [1]. Two types of *Salmonella* can be distinguished based on their host adaptability and infectious nature: human typhoidal and non-typhoidal *Salmonella* (NTS). Typhoidal *Salmonella,* associated with typhoid fever, is host-restricted and infects humans. It disseminates systematically and colonizes the gallbladder, and infected individuals become chronic carriers. In contrast, NTS, primarily associated with enteric infections, has a broad host range, that includes chickens, cattle, pigs, humans, and other species [2]. NTS is shed intermittently for a short period of time and depends on the environment for its survival and transmission [3]. The clinical presentation of NTS includes gastroenteritis and diarrhea [4]. However, *Salmonella* can disseminate from the intestine to other systemic organs during invasive infections. Based on surface antigens, there are >2600 serotypes of *Salmonella* [5], with invasive infections primarily caused by the serotypes *S.* Typhimurium, *S.* Enteritidis, and *S.* Dublin [6]. NTS is the second leading cause of death associated with foodborne illnesses in the USA, with almost 420 deaths reported yearly, causing huge public health impacts [7]. NTS accounted for losses of USD 3.3 billion in terms of treatment and human productivity in the United States due to high morbidity and mortality rates [8].

The difficulty of treating human salmonellosis is associated with the evolution of AMR genes in *Salmonella*. The extensive use of antibiotics, such as for prophylaxis and metaphylaxis in food-animal production systems, is accelerating the evolution of AMR in *Salmonella*. For instance, *Salmonella* in chickens treated with ceftiofur for *Salmonella* infections has become resistant to ceftiofur [9]. Similarly, the use of high levels of tetracycline and sulfonamide on poultry farms in Nigeria was positively correlated with tetracycline and sulfonamide resistance in *Salmonella* [10]. Likewise, tetracycline, streptomycin, and nalidixic acid resistance were observed in *Salmonella* isolated from poultry farms in the southeastern United States [11]. In 2018–2019, azithromycin- and ciprofloxacin-resistant *S.* Newport was isolated from a dairy farm in the USA [12]. The consumption and improper handling of farm products and direct or indirect contact with the animals on farms pose a risk for the transfer of antibiotic-resistant *Salmonella* to humans, which can reduce the effectiveness of drugs and increase the rates of hospitalization and mortality in humans [13]. Therefore, the Centers for Disease Control (CDC) and the World Health Organization (WHO) have prioritized the improved monitoring of AMR *Salmonella* along the food chain, particularly in food-producing animals.

Farm animals infected with *Salmonella* can repeatedly shed bacteria in their feces, contaminating the farm environment [14,15]. Thus, contaminated farm water and raw manure serve as vehicles for *Salmonella* transmission to farm produce [16]. *Salmonella* can be internalized by produce grown in a field irrigated with contaminated water or amended biologically with contaminated manure [17]. Hence, farm environments could be a source of indirect transmission of *Salmonella* to humans. In previous studies, he prevalence of *Salmonella* was 15.2% in poultry manure [18], 4.9% in dairy manure [19], and 29.4% in pond water used for irrigation [20]. Once present in the farm environment, *Salmonella* can form biofilms to persist for a long time [21]. Similarly, Kuang et al. detected *S.* Typhimurium and *S.* Enteritidis in farm animals in China, which are the two most important serotypes predominantly reported in human infections [22].

Small specialty crop farms (SSCFs) are an important sector of crop production in the United States [23,24]. The SSCF enrolled in our study are mixed crop-and-animal farm systems where livestock/poultry are grown close to the farm produce in the same facility. Produce from SSCFs is considered fresh, healthy, and safe for consumption [25]. Therefore, it is sold directly to consumers in farmers’ markets, stores, or roadside stands and sometimes to grocery stores [25]. SSCFs are family-owned and have their own practices, that differ between farms. Biological soil amendments of animal origin (BSAAOs), such as poultry and dairy manure, are commonly used as biofertilizers on these SSCFs. Untreated irrigation water is used to irrigate fields. Differences in farming practices, such as the application of BSAAOs and the use of irrigation water potentially contaminated with infected farm animal feces, increase the risk of foodborne pathogens in produce grown on these farms. Similarly, antibiotics are used on SSCFs to enhance growth and prevent and treat diseases in animals [26,27]. The unregulated use of antibiotics can lead to the emergence of multidrug-resistant *Salmonella* [26,27]. Differences in farming practices, such as the application of BSAAOs, the use of untreated irrigation water, and the indiscriminate use of antibiotics, increase the potential risk of the transmission of antibiotic-resistant foodborne pathogens from SSCFs to humans.

While NTS cause a self-limiting disease, antibiotics are needed to treat invasive infections. Recently, quinolones, fluoroquinolones, and third-generation cephalosporins have been used to treat salmonellosis, with colistin used as a last-resort drug for bacterial infections [28]. The emergence and dissemination of MDR *Salmonella*, including colistin-resistant isolates from food-animal production farms, are limiting the choice of therapeutic treatment for salmonellosis [29]. Graham et al. reported the possibility of a spillover of AMR genes from SSCFs to humans [27]. Although AMR genes can arise in *Salmonella* from chromosomal mutations, the primary mechanism to acquire them is through plasmids [21]. Plasmids can additionally harbor virulence genes that enhance the pathogenesis of *Salmonella*, including mechanisms for host immune evasion, attachment, entry, colonization, and invasive and persistent infection. Several virulence genes, such as *cdtB*, *rck*, *sodCI*, *pef*, and *spv*, were detected in *Salmonella* samples from poultry farms [30].

Although several studies have performed comparative genomic analyses of *Salmonella* isolated from commercial food-animal production systems, here, we examined the serotypes, resistome, virulome, and plasmidome of *Salmonella* recovered from SSCFs [31,32], which have not been reported previously. For instance, we characterized the serotypes of *Salmonella* to detect the serotypes important for human infection, assessed the resistome and virulome to determine AMR genes and virulence genes, and identified the plasmids and their colocalization with AMR and virulence genes to understand the potential for horizontal transmission. With an increasing number of people relying on locally grown produce, it is important to understand the potential public health risks posed by such farms. Therefore, this study utilized whole-genome sequencing (WGS) to assess the genomic diversity among the isolates using pan-genome analysis and phylogenetics to identify ARGs and virulence genes and to characterize the plasmid profiles of *Salmonella* recovered from SSCFs. In addition, phenotypic assays, such as antimicrobial susceptibility testing and biofilm assays, were performed to associate the results with the genotypic data.

## 2. Results

### 2.1. Genomic Features of Salmonella Revealed 15 Serotypes Belonging to 18 Sequence Types with 3436 Core Genes

In our study, conducted between 2016 and 2021, 2.27% of samples were found to be positive for *Salmonella*. *Salmonella* was isolated from four different sources, dairy manure, poultry manure, soil, and water samples, with prevalence of 2.63%, 2.85%, 0.18%, and 8.12%, respectively (Table 1). The number of samples collected from each farm and the frequency of collection during the study period are provided in Table S1. This study is an extension of our earlier published study in which, *Salmonella* prevalence was further discussed [24]. From these 29 samples, a total of 80 isolates were sequenced. Multiple isolates per samples were sequenced because studies have shown that multiple serotypes can co-exist in the same sample [33]. Two assemblies were discarded due to low genomic coverage, and subsequent analyses were therefore carried out with the remaining 78 isolates. Summary statistics for the genome assemblies are given in Table S2, and serotype averages are provided in Table 2. Overall, the average genomic depth of coverage was 64×, the GC content was 52.18%, the genome size was 4.6 Mbp, and the number of contigs was 43.

Both serotyping and sequence typing were used to classify *Salmonella* isolated in this study. In silico serotyping using SeqSero2 resulted in the detection of 15 serotypes (Table 3), with *S.* Cerro being the most common, accounting for 29.4% of genome assemblies. Notably, 8 out of the 15 serotypes reported in our study are among the top 20 serotypes frequently reported in human salmonellosis [34].

We detected 18 unique sequence types (STs) (Table 3 and Table S2). Twelve serotypes were associated with one ST, while three serotypes had two STs: *S.* Cerro (associated with ST367 and ST9243), *S.* Montevideo (associated with ST4 and ST138), and *S.* Newport (associated with ST45 and ST350). To the best of our knowledge, this is the first study to detect ST9243 in *S.* Cerro.

**Figure 1:**
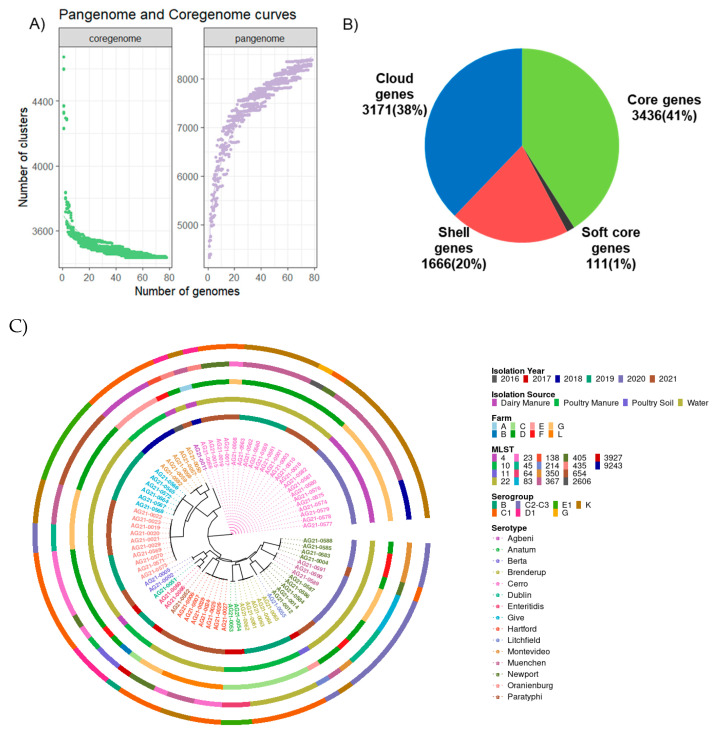
(**A**) Rarefaction curves for core (3436) and pan-genes (8384) of the 78 isolates. The *x*-axis represents the number of genomes. (**B**) Pie chart showing the pan-genome composition of the isolates. Core genes are present in 100% of isolates, with soft-core genes in 95–99%, shell genes in 15–99%, and cloud genes in <15% of isolates. (**C**) Whole-genome core SNP-based phylogeny of *Salmonella enterica* isolates (*n* = 78), with samples (tips) colored by serotype. Each isolate is color-coded differently in five different circles based on the isolation year, isolation source, farm type, MLST, and serogroup.

A pan-genome analysis showed that 41% of genes were core genes, 1% were soft-core genes, 20% were shell genes, and 38% were cloud genes (Figure 1A,B). The accessory genes consist of soft-core genes, shell genes, and cloud genes. Functional annotation of core genes showed that they were mostly involved in metabolic processes such as transcription, translation, cell wall synthesis, energy production, and carbohydrate, amino acid, and lipid transport (Figure S1A). The majority of accessory genes were mobility elements, such as prophages and transposons (Figure S1B). Our finding that core genes represented less than half of all genes was due to the presence of distinct sets of genes among the 15 serotypes of *Salmonella* and implies relatively high genomic variability among the serotypes.

### 2.2. A phylogenetic Tree Showed That All Serotypes except S. Newport form Monophyletic Clades

The genetic similarity of the isolates was first computed using average nucleotide identity (ANI) values. The ANI values were between 98 and 100%, which indicates that the isolates were highly similar. Furthermore, a phylogenetic tree was inferred using the core SNPs identified by kSNP3. The optimal k-mer size identified by kSNPS’3 Kchooser was 19 bp (thus, each putative SNP was taken as the central base in a 19-mer). A total of 170,689 SNPs were detected, of which 60,089 were core SNPs. A phylogenetic tree showed that samples assigned to the same serotype formed monophyletic clades, except for *S.* Newport, which was found across two clades along with *S.* Muenchen and *S.* Litchfield (Figure 1C).

Interestingly, six serotypes (*S.* Cerro, *S.* Montevideo, *S.* Enteritidis, *S.* Oranienburg, *S.* Hartford, and *S.* Newport) were found on more than one farm, and multiple serotypes were detected on four farms, suggesting that *Salmonella* lineages from multiple sources present on one farm also circulate on other farms. To explore the effects of geographical distance on the movement of *Salmonella*, a multi-dimensional scaling (MDS) plot was constructed to visualize the distances between farms (Figure S2). Our results showed that farms D, F, and G clustered together. *S.* Cerro was found on farms A, D, F, and G, *S.* Newport was found on farms D and F, and *S.* Oranienburg was found on farms D and G. Therefore, due to the close proximity of the three farms D, F, and G, *S.* Cerro, *S.* Newport, and *S.* Oranienburg might have been disseminated from one farm to another. Likewise, the phylogenetic tree showed that five serotypes were recovered from a single farm only (excluding the four serotypes with only one sample): *S.* Anatum (farm C), *S.* Braenderup (C), *S.* Give (D), *S.* Berta (D), and *S.* Muenchen (G).

### 2.3. Pan-Genome-Wide Association Study (Pan-GWAS)

**Figure 2:**
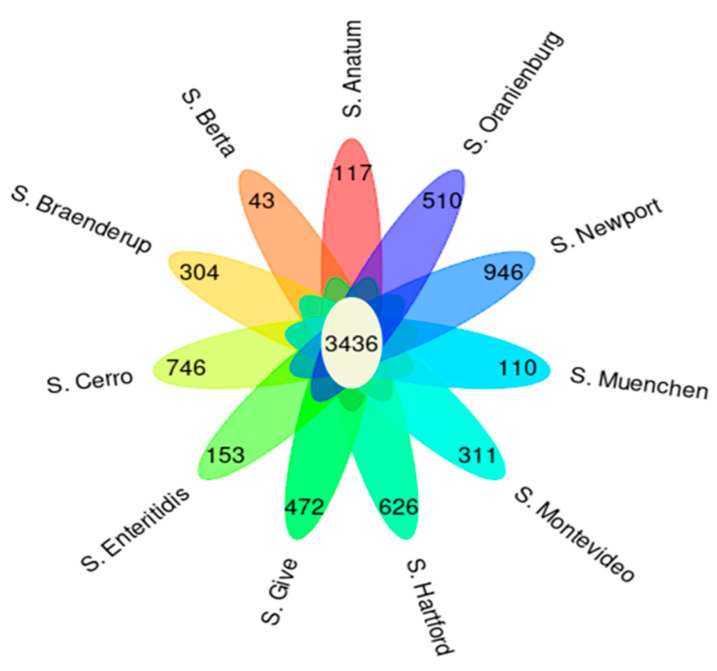
Floral Venn diagram showing the number of orthologous genes present in all isolates in the center of the flower as core genes (3436). The petals represent positively associated genes with serotype based on Scoary results.

Among 4948 accessory genes, Scoary detected 1172 genes that were significantly associated with a total of eleven serotypes: *S.* Anatum (117), *S*. Berta (43), *S.* Braenderup (304), *S.* Cerro (746), *S.* Enteritidis (153), *S.* Give (472), *S.* Hartford (626), *S.* Montevideo (311), *S.* Muenchen (110), *S.* Newport (946), and *S.* Oranienburg (510) (Figure 2). These significantly associated genes included the macrolide export protein in *S.* Cerro, a mercury resistance protein in *S.* Newport, and a phosphotransferase protein in *S.* Give (Table S3). The significance of the associations could not be calculated for the four serotypes containing only one sample per serotype.

### 2.4. Antimicrobial Resistance Genes and Detection of the ACSSuT Cassette

The resistome analysis detected the presence of eight unique antimicrobial resistance genes (ARGs) in five serotypes of *Salmonella* that can confer resistance to drugs within seven antibiotic classes (Table 4). The AMR genes detected were *fosA7*, *blaCMY-2*, *sul2*, *aph(3″)-Ib*, *aph(6)-Id*, *tet(A)*, *floR*, and *gyrA_D87Y*. Seventy-five percent of isolates with ARGs were MDR. *S.* Montevideo and *S.* Enteritidis harbored one ARG, *S.* Muenchen harbored three ARGs, and *S.* Dublin and *S.* Newport harbored five ARGs. Interestingly, *S.* Dublin and *S.* Newport harbored the ACSSuT cassette, which confers resistance to ampicillin, chloramphenicol, streptomycin, sulfonamides, and tetracyclines, on a plasmid [35]. The beta-lactamase resistance gene, which confers ampicillin resistance [36], was detected in *S.* Dublin and *S.* Newport. Likewise, the SSuT resistance pattern was observed in *S.* Muenchen. The screening of genomic mutations showed the presence of a single nonsynonymous mutation at the 87th amino acid of the chromosomal quinolone-resistance-determining region (QRDR) in *S.* Enteritidis. The change from aspartic acid to tyrosine at the 87th position of the gyrase genes confers resistance to quinolones, which is the most detected mutation in nalidixic acid-resistant *Salmonella* [37].

### 2.5. Phenotypic Antimicrobial Susceptibility Testing Revealed Colistin Resistance in Some Isolates

**Figure 3:**
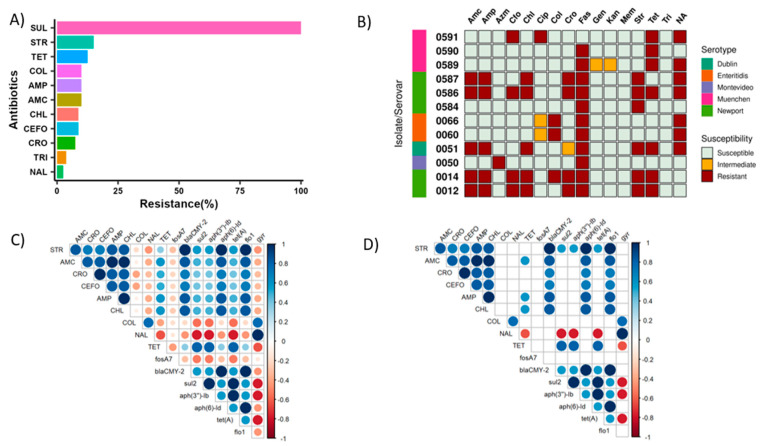
(**A**) Summary of overall resistance percentages of *Salmonella* isolates (*n* = 80) to 16 different types of antibiotics determined by broth microdilution method. The data shown in the bar graph are averages across two independent experiments. The minimum inhibitory concentrations (MICs) of the antibiotics (μg/mL) tested were as follows: streptomycin (STR: 32–64), kanamycin (KAN: 16–64), gentamycin (GEN: 4–16), amoxicillin (AMC: 8–32), ceftriaxone (CRO: 1–4), cefoxitin (CEFO: 8–32), sulfisoxazole (SUL: 256–512), trimethoprim-sulfamethoxazole (TRI: 2–4), azithromycin (AZM: 16–32), meropenem (MEM: 1–4), ampicillin (AMP: 8–32), chloramphenicol (CHL: 8–32), colistin (COL: 2–2), ciprofloxacin (CIP: 0.06–1), nalidixic acid (NAL: 16–32), and tetracycline (TET: 4–16). (**B**) Phenotypic AST results of the isolates possessing AMR genes. (**C**,**D**) Correlation matrix of phenotypic (antibiotic resistance) and genotypic (antibiotic resistance genes) features showing all correlations (**C**) and significant (*p* < 0.05) correlations (**D**). Circles are only shown for significant correlations (**D**), where circle size indicates the strength of the correlation, and circle color indicates the sign of the correlation: blue circles indicate a positive correlation, and red ones represent a negative correlation. Antibiotics (phenotypic resistance) are listed first with capitalized names, and ARGs (genotypic resistance) are listed next and have all-lowercase names.

The overall resistance percentages according to phenotypic antimicrobial susceptibility testing (AST) for all isolates are shown in Figure 3A. Surprisingly, all 80 isolates were resistant to sulfisoxazole (100%), and resistance to streptomycin was the next most common (15%), followed by tetracycline (12.5%) and colistin (10%). Colistin is a last-resort antibiotic used to treat infections with MDR Gram-negative pathogens. Despite being banned for clinical use and animal production in the USA due to concerns about toxicity, we found the colistin-resistant phenotype in eight isolates [38]. Similarly, we detected resistance to amoxicillin (10% of isolates), ampicillin (10%), trimethoprim–sulfamethoxazole (3.75%), ceftriaxone (7.5%), cefoxitin (8.75%), chloramphenicol (8.75%), and nalidixic acid (2.5%). None of the isolates were resistant to azithromycin, ciprofloxacin, gentamycin, kanamycin, or meropenem. We also found that 26.25% of isolates were resistant to two different classes of antibiotics, while 13.75% were MDR.

### 2.6. Association between Phenotypic and Genotypic Antibiotic Resistance

A correlation analysis was used to examine the associations between phenotypic and genotypic (eight ARGs) antibiotic resistance (Figure 3C,D). A significant negative correlation was observed between the presence of the tetracycline resistance gene and the nalidixic-resistant phenotype (Figure 3D, *p* < 0.05). Positive correlations were found between several ARGs and resistance phenotypes (Figure 3D, *p* < 0.05), such as between the presence of tetracycline gene and tetracycline resistance (Figure 3D, *p* < 0.05). Similarly, the presence of the beta-lactamase gene was positively correlated with β-lactam amoxicillin resistance, the chloramphenicol gene was positively correlated with chloramphenicol resistance, and the streptomycin resistance genes were positively correlated with streptomycin resistance (Figure 3D, *p* < 0.05).

We also observed significant correlations between the occurrences of ARGs themselves: for instance, there was a significant negative correlation between the presence of gyrase gene (which confer resistance to quinolones) and tetracycline resistance gene, whereas there was a significant positive correlation between streptomycin resistance genes and amoxicillin, ampicillin, and chloramphenicol resistance genes (Figure 3D, *p* < 0.05).

### 2.7. Plasmid Profile Revealed the Presence of Nine Plasmid Replicons

**Figure 4:**
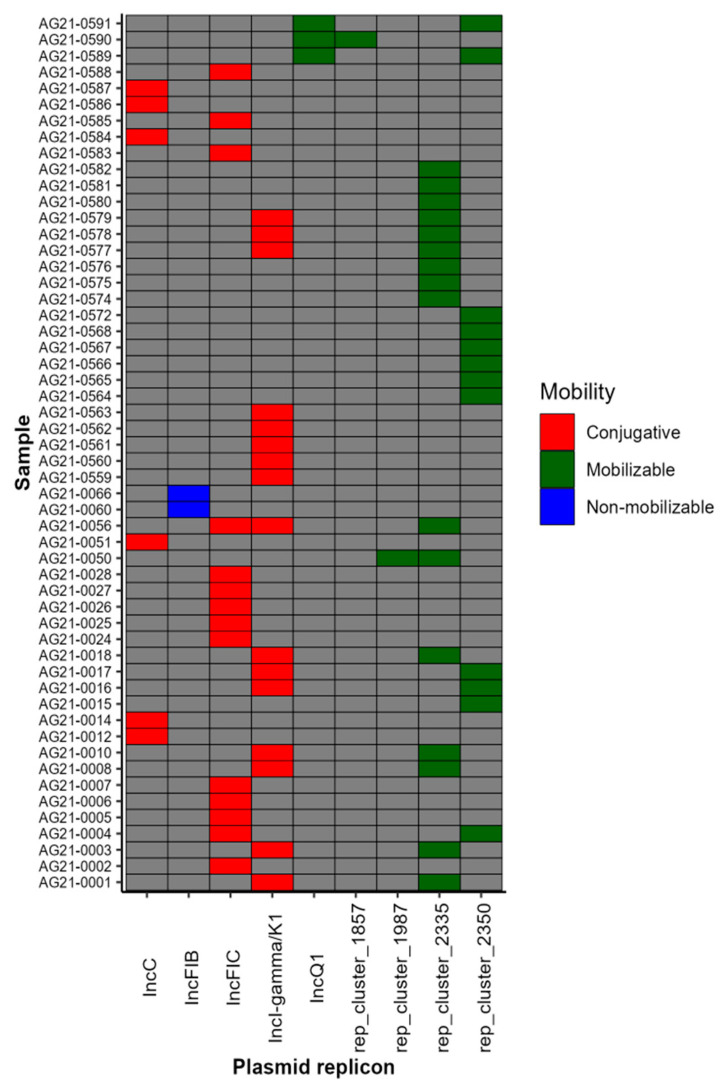
Heatmap showing the presence of conjugative, mobilizable, and non-mobilizable plasmids among the isolates. The MOB-suite package was used to determine the plasmid replicon, plasmid mobility, and size of the plasmid (Table S4).

To understand the genetic basis of ARG dissemination, we investigated the presence of plasmids. A total of nine distinct plasmid replicons were detected, with 69% (54/78) of our isolates containing at least one plasmid (Figure 4). Among isolates with plasmids, different types of incompatibility groups (IncC, IncI-gamma/K1, IncFIB, IncFIC, and IncQ1) were detected in 46% (25/54), rep plasmids were detected in 26% (14/54), and Inc and rep plasmids were detected in 28% (15/54) of the isolates. Additionally, conjugative plasmids were present in 42.6% (23/54), mobilizable plasmids were present in 31.5% (17/54), and non-mobilizable plasmids were present in 3.7% (2/54) of the isolates (Table S4). Some samples harbored more than one plasmid: 22.2% (12/54) contained both conjugative and mobilizable plasmids. Similarly, the occurrence of ARGs on plasmid contigs was evaluated, and we found that 75% of ARGs were assigned to be located on plasmid contigs. Interestingly, the IncC conjugative plasmid replicon was reported on the same contig as the ACSSuT resistance pattern genes in *S.* Newport and *S.* Dublin. Likewise, the SSuT resistance pattern in *S.* Muenchen was detected on the same contig as the IncQ1 mobilizable plasmid replicon.

### 2.8. S. Enteritidis and S. Dublin Have Unique Virulence Genes

**Figure 5:**
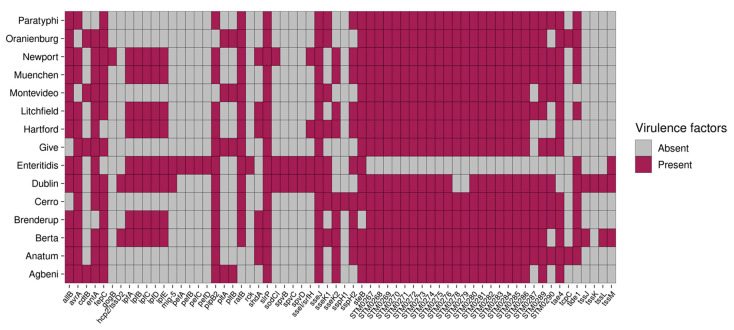
Variable virulence genes identified and detected by ABRicate and the Virulence Factor Database (VFDB).

Three types of secretion systems (T1SS, T3SS, and T6SS) were located in two *Salmonella* pathogenicity islands, and one *Salmonella* centrosome island was observed. A total of 176 unique virulence genes were detected, 112 of which were shared among all isolates (Table S5). These genes represent the core virulome, likely needed for the survival of *Salmonella*. The remaining 62 genes were not present in all serotypes (variable virulome, Figure 5). Two genes were present in all serotypes but absent in a few individual isolates: *slrP* was present in 69 isolates, and *sseJ* was found in 72 isolates.

Virulence genes that were found only in a subset of serotypes included the following. First, the *spv* genes located on the *Salmonella* virulence plasmid (pSV), which is involved in survival and systemic infection in host cells, were detected in *S.* Enteritidis and *S.* Dublin [39]. The *spvABCD* genes are arranged in an operon and are positively regulated by the upstream *spvR* gene [39]. However, *spvR*, which is needed for the regulation of the *spv* locus [40], was absent in our isolates. Second, long polar fimbriae genes (*lpfABCDE*) and plasmid-encoded fimbriae genes (*pefABCD*) located on the pSV were detected in *S.* Enteritidis. The serum resistance gene (*rck*), which increases the invasion of host cells and the resistance to killing mediated by the complement system, was detected in *S.* Enteritidis. The presence of *mig5*, *rck*, *spv*, and *pef* genes in *S.* Enteritidis suggests that these genes play an important role in the pathogenesis of human salmonellosis [41]. Likewise, *lpfABCDE* was detected in other serotypes, such as *S.* Braenderup, *S.* Dublin, *S.* Muenchen, and *S.* Newport. Finally, the *cdtB* gene, which produces the cytolethal distending toxin (CDT), was detected in *S.* Agbeni, *S.* Give, *S.* Oranienburg, and *S.* Montevideo. However, *cdtA* and *cdtC* genes, which are required for the binding of the CDT to target cells, were not detected. Pertussis-like toxins (*pltA* and *pltB*), which share structural and functional homology with *cdtA* and *cdtC* genes, forming a complex with *cdtB* and delivering it from the intracellular compartment to target cells, were detected in isolates with *cdtB* [42].

### 2.9. S. Give Is a Strong Biofilm Producer

**Figure 6:**
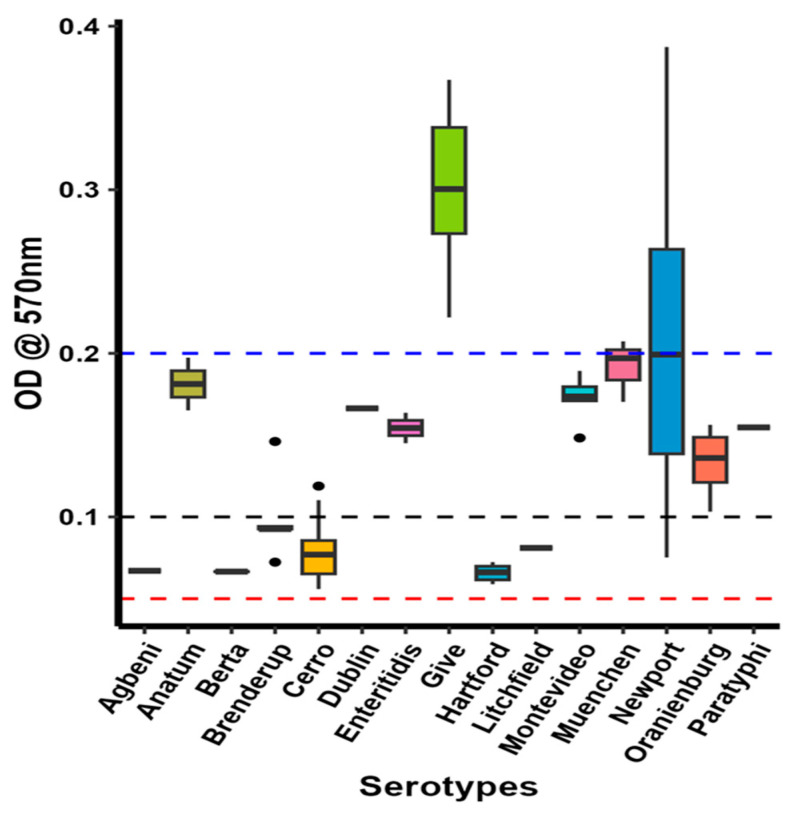
Biofilm formation ability of the isolates, as determined by crystal violet staining. Isolates between the red (OD = 0.05) and the black (OD = 0.10) lines are weak biofilm producers, those between the black and the blue (OD = 0.20) lines are moderate biofilm producers, and those above the blue line are high biofilm producers.

The results of the phenotypic biofilm production assay are shown in Figure 6. Five serotypes, *S.* Agbeni, *S.* Berta, *S.* Braenderup, *S.* Hartford, and *S.* Litchfield, were low biofilm producers. Most *S.* Cerro isolates were low biofilm producers, whereas a few were moderate biofilm producers. *S.* Anatum, *S.* Dublin, *S.* Enteritidis, *S.* Montevideo, *S.* Muenchen, *S.* Oranienburg, and *S.* Paratyphi were moderate biofilm producers. A few *S.* Newport and *S.* Give isolates were high biofilm producers, whereas other *S.* Newport isolates were moderate biofilm producers.

### 2.10. Comparative Genomic Analysis Revealed SSCFs as Potential Reservoirs for AMR Gene Transmission to Humans

**Figure 7:**
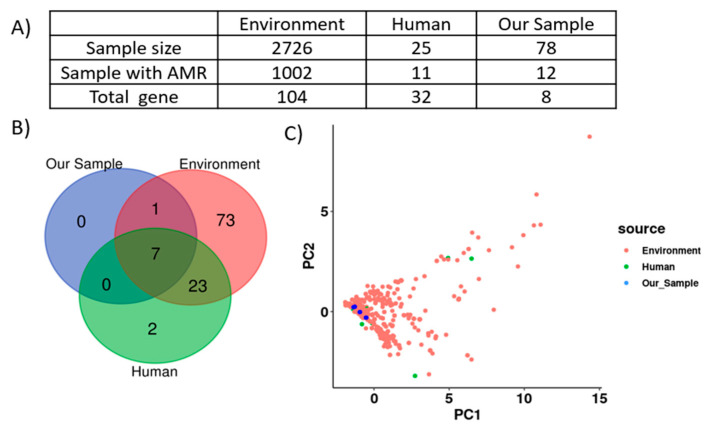
A comparison of AMR gene profiles of *Salmonella* isolates from this study with those deposited in NCBI. (**A**) Table with the details of the number of the isolates downloaded and their AMR genes, including our isolates. The downloaded isolates from Ohio were classified as either “environment” or “human” based on the source of their isolation. (**B**) Venn diagram showing the number of AMR genes shared between our samples and human and environmental isolates. (**C**) PCA of presence/absence of antibiotic resistance genes, with each dot representing an individual isolate.

A total of 2751 *Salmonella* genomes sampled in Ohio were downloaded from NCBI. Among them, AMRFinderPlus detected 104 unique ARGs in 1002 environmental samples and 32 ARGs in 25 human samples (Figure 7A). Among them, seven ARGs were common to humans, the environment, and our *Salmonella* isolates (Figure 7B). Further, principal component analysis (PCA) showed an overlap of ARG profiles between our isolates and *Salmonella* isolates reported to be from humans and the environment in Ohio (Figure 7C), suggesting the potential for the transmission of drug-resistant *Salmonella* from SSCFs to humans.

## 3. Discussion

Numerous studies have linked salmonellosis outbreaks to food-animal farms [43,44,45]. Clinically and sub-clinically infected farm animals regularly shed *Salmonella*, thereby contaminating the farm environment. *Salmonella* can survive outside its host in farm environments such as manure, soil, and water for 184, 332, and 1825 days, respectively [46,47]. The survival or persistence of *Salmonella* in farm environment poses difficulties in removing *Salmonella* from the farm due to the possibility of reinfection or reinoculation [48]. BSAAOs are commonly applied as biofertilizers on SSCFs. The application of BSAAO can contaminate the soil because *Salmonella* can survive in the manure-amended soil for up to 329 days. This increases the risk of *Salmonella* internalization by the produce grown in the field and transmission to humans from the food production chain. Usegi et al. reported that *S.* Enteritidis phage type (PT) 30 associated with an outbreak from 2000 to 2001, persisted in an almond orchard for 5 years [49].

Despite evidence of *Salmonella* outbreaks originating from farms, none of the previous studies have compared *Salmonella* genomes isolated from SSCFs [50]. Our analysis identified 15 serotypes of *Salmonella* circulating on eight SSCFs, suggesting the presence of diverse *Salmonella* in this niche. However, all *Salmonella* serotypes detected on SSCFs are not equally important for human infection. For instance, *S.* Cerro is mostly associated with infection in cattle, whereas the serotypes *S.* Enteritidis and *S.* Newport are frequently reported in human infections. The most prevalent serotype was *S.* Cerro ST367, which was recovered from water and dairy manure. This result is consistent with previous studies in which *S.* Cerro was predominantly isolated from dairy farms [32]; however, the presence of this serotype is not as clinically concerning because *S.* Cerro is a cattle-adapted serotype that is rarely isolated from humans and is usually limited to gastrointestinal infections [32,51,52]. However, our study also detected two clinically important serotypes, *S.* Enteritidis and *S.* Newport, which are the serotypes most frequently reported in human salmonellosis in the USA [34]. The prevalence of clinically important serotypes of *Salmonella* on SSCFs necessitates the implementation of strict biosecurity measures to reduce their transmission to humans. A correlation analysis was carried out to detect an association between the presence of specific serotypes and specific farm practices, such as the application of either poultry or dairy manure. However, we did not see any statistically significant correlations (*p* > 0.05) between the presence of certain serotypes and manure application.

The serotypes *S.* Anatum, *S.* Berta, *S.* Braenderup, *S.* Give, and *S.* Muenchen were recovered from only a single farm. In contrast, the serotypes *S.* Cerro, *S.* Enteritidis, *S.* Hartford, *S.* Montevideo, *S.* Newport, and *S.* Oranienburg were recovered from multiple farms. The three farms from which *S.* Cerro, *S.* Newport, and *S.* Oranienburg were isolated were in close geographical proximity, suggesting the transmission of serotypes between farms. The presence of the same serotypes on multiple farms was also reported in a previous study [53]. It is likely that these serotypes are circulating via horizontal transfer through the wind, equipment, vehicles, or humans. SSCFs share their farm equipment, between farms, which may be the potential route of *Salmonella* transmission. The horizontal transmission of *Salmonella* between dairy farms through tractors was reported in England by Brennan et al. [54].

The analysis of core-genome SNPs is a widely used method for constructing phylogenetic trees and investigating outbreaks [55]. In our phylogenetic analysis, most *Salmonella* serotypes were monophyletic, except for *S.* Newport, which was found in two separate clades. An ANI analysis showed no differences between the two strains of *S.* Newport, with 99–100% similarity between the two *S.* Newport strains. However, the two *S.* Newport strains showed differences in their sequence types [56]. One clade was composed of ST45, while the other was made up of ST350. ST45 contained AMR genes and mercury resistance genes, whereas ST350 did not carry any resistance genes. In addition, differences in plasmids content were detected between ST45 and ST350. Since ST45 is more frequently associated with outbreaks of *Salmonella* in humans, it poses a serious public health concern [57,58]. However, there are no reports on ST350 in outbreaks of *S.* Newport. Differences in the prevalence of the AMR and heavy-metal-resistance genes in ST45 of *S.* Newport could also be related to different selection pressures among farms. However, we did not observe any association between the farm identity and the STs of *S.* Newport.

Our study revealed the presence of eight unique AMR genes in five *Salmonella* serotypes on fourteen farms. The factors that might contribute to differences in the prevalence of different AMR genes in these farms are unknown. The SSCFs are family-owned and have management practices, that differ between individual farms. We hypothesize that management practices, for example, different uses of antibiotics and other antimicrobials, such as biocides and disinfectants, which co-select AMR genes with antibiotic resistance genes [29], may have contributed to the differences in resistance genes among *Salmonella* isolates. The majority of *Salmonella* isolates in our study were resistant to sulfisoxazole (100%), streptomycin (15%), and tetracycline (12.5%), which are the first-line drugs used for the treatment of salmonellosis. Similar to our study, 90% of *Salmonella* recovered from a hatchery were resistant to sulfisoxazole [59]. In 1990, when *Salmonella* became resistant to first-line antibiotics such as chloramphenicol, ampicillin, and trimethoprim–sulfamethoxazole, quinolones and fluoroquinolones became the preferred antibiotics for treating *Salmonella* infections [60]. Resistance to quinolones is attributed to mutations in genes such as *gyrA*, *gyrB*, *parC*, and *parE* [61]. In our study, we detected a mutation in the A subunit of the gyrase gene of *S.* Enteritidis that changed the amino acid aspartate to tyrosine at the 87th position, conferring resistance to quinolones in 2.5% of the isolates [62]. In addition, the phenotypic AST data showed that *S.* Enteritidis was resistant to colistin, a last-resort antibiotic used for treating multidrug-resistant Enterobacteriaceae infections [63]. Moreover, a colistin-resistant phenotype was observed in serogroups B, C2-C3, D, K, and E1, which accounted for 10% of our isolates. The occurrence of colistin resistance in *S.* Enteritidis was unsurprising because *Salmonella* serotypes belonging to serogroup D have a higher tendency to develop colistin resistance [29]. The absence of plasmid-mediated mobile colistin resistance (*mcr*) genes in the isolates reduces the chances of horizontal transmission of colistin resistance genes [64]. However, an examination of the protein sequences of genes that have been reported for colistin resistance in *Salmonella* (such as *pmrAB*, *LolB*, *mgrB*, and *phoPQ*) revealed two nonsynonymous mutations in the *LolB* gene of *S.* Cerro: P25L and S194N. The presence of these mutations is concerning for public health because colistin-resistant *Salmonella* can be potentially transmitted to humans from SSCFs. This will reduce the effectiveness of colistin, which is a last-resort antibiotic for bacterial infections. Olaitan et al. reported the horizontal transmission of colistin-resistant *E. coli* carrying unknown mutation from pigs to humans [65,66]. Likewise, colistin is used at sub-MIC concentrations in combination with other drugs to enhance its effectiveness [65]. However, the synergistic effect will not be observed in the presence of *Salmonella* carrying mutations in colistin resistance genes. The mutation in *LolB* was absent in colistin-susceptible *S.* Cerro. The *LolB* gene is involved in the transport of lipoproteins to the outer membrane [29]. We speculate that the mutation in the *LolB* reduces the transport of the lipoprotein to the outer membrane, which decreases the negative charge of the bacteria. Colistin is a positively charged cationic peptide that interacts with the negatively charged bacterial membrane [64]. The modification in the outer membrane of *S.* Cerro may reduce the interaction of colistin with the bacterial membrane, thereby conferring resistance. The absence of colistin-resistance-conferring mutations and plasmid-mediated colistin resistance genes in other serotypes suggests the presence of a novel colistin resistance mechanism, which needs further study.

An MDR ACSSuT cassette was detected in *S.* Dublin and *S.* Newport, while an SSuT cassette was detected in *S.* Muenchen. Consistent with our study, mobilizable IncQ1 plasmids with the SSuT resistance pattern have been reported in *S.* Typhimurium [67]. These results suggest that the *Salmonella* isolates characterized in this study are potential reservoirs for the transmission of MDR ARGs to humans. This finding was further corroborated by PCA analysis, which showed that our isolates clustered with environmental and human isolates (Figure 7C). This implies that *Salmonella* isolates recovered from humans and environmental sources carried similar sets of ARGs to those detected in our isolates. This represents a serious public health issue because MDR *Salmonella* can increase the rate of hospitalization, invasive illness, and mortality globally [68].

Plasmids have been reported to play a significant role in the dissemination of AMR genes, virulence genes, and other traits conferring a fitness advantage. Our study detected nine different plasmid replicons, predominantly (97%) belonging to either conjugative or mobilizable plasmids, suggesting that *Salmonella* frequently acquires genes via the horizontal transfer of plasmids. The IncC conjugative plasmid, which carried the highest number of AMR genes, was detected only in *S.* Newport and *S.* Dublin. Consistent with our results, a previous study found the conjugative IncC plasmid in *S.* Newport [69]. Interestingly, the IncC (broad host) conjugative plasmid replicon was found on the same contig as the ACSSuT cassette in our study. This implies that the IncC plasmid may be disseminating MDR genes within *Salmonella* and other genera in the Enterobacteriaceae family through conjugation. This is particularly concerning because several studies have detected IncC conjugative plasmids in clinical isolates of pathogenic *Salmonella* and *E. coli* [70,71]. The acquisition of the ACSSuT cassette will make *Salmonella* resistant to multiple antibiotics and limit the treatment options for salmonellosis.

Virulence genes in *Salmonella* are important for its fitness, such as persistence in a specific niche and successful pathogenesis. In this study, we identified the *shdA* gene, which encodes the nonfimbrial adherence gene, only in isolates recovered from water, poultry manure, or soil amended with poultry manure. This gene is involved in the persistent colonization and long-term fecal shedding of *Salmonella* [72]. The *shdA* gene was detected in six serotypes: *S.* Anatum, *S.* Berta, *S.* Braenderup, *S.* Hartford, *S.* Litchfield, and *S.* Newport. Previous studies have shown the presence of *shdA* in *S.* enterica adapted to warm-blooded animals such as mice, poultry, and pigs [72,73,74]. However, the absence of *shdA* among isolates recovered from the dairy manure in our study needs further exploration, for example, to determine whether this gene is present in cattle-adapted *Salmonella*. Similarly, multiple virulence genes that enhance the pathogenesis of *Salmonella* serotypes were detected in our study. The *SpvBCD* operon, which enhances the intracellular proliferation of *Salmonella* in human cells and causes invasive infections, was detected in *S.* Enteritidis and *S.* Dublin [75]. In *S.* Enteritidis, it was associated with non-mobilizable IncFIB plasmids, whereas in *S.* Dublin, it was found on an IncC conjugative plasmid. Consistent with our study, previous studies have shown the presence of the *spv* operon in *S.* Dublin and *S.* Enteritidis [76,77]. Furthermore, the *cdtB* gene, in association with *pltA* and *pltB* genes, which encodes for a typhoid toxin that arrests the cell cycle, was detected in *S.* Agbeni, *S.* Give, *S.* Oranienburg, and *S.* Montevideo [78]. Earlier studies have reported *cdtB* genes in all four serotypes [79,80,81]. Although these four serotypes do not represent a concern as MDR bacteria, the virulence genes pose a potential health risk.

To investigate whether certain *Salmonella* serotypes may be better adapted to farm environments by forming biofilms, we measured the biofilm production capacity of all isolates. Studies have shown that high-biofilm-producing *Salmonella* serotypes are resistant to antimicrobials, disinfectants, and thermal and environmental stress such as desiccation [82,83]. This resistance allows them to survive in harsh farm environment conditions and cross-contaminate farm produce, which can lead to their transmission to humans through the food production chain. Our results showed that most serotypes were moderate biofilm producers. Notably, *S.* Give and a few isolates of *S.* Newport were strong biofilm producers (Figure 6). A correlation analysis was conducted between phenotypic biofilm formation ability and the biofilm-associated genes present in these two serotypes. However, we did not see any significant correlation (*p* > 0.05) between the phenotypic data and the presence or absence of biofilm-associated genes. Several studies have reported *S.* Newport as a high biofilm producer and suggested that biofilm formation is a strategy employed by *S.* Newport to survive in harsh environmental conditions [84,85]. However, here, we also report a high biofilm formation ability in the *S.* Give serotype, which has not been described previously. Interestingly, the Scoary results demonstrated a significant association between *S.* Give and several biofilm-related genes (Table S2). For instance, the DNA adenine methylase gene (*dam*) was significantly associated with *S.* Give, *S.* Newport, and *S.* Montevideo. This gene regulates the expression of the *csgD* gene, which is involved in the formation of curli and cellulose, important components of biofilms [86]. Furthermore, the Scoary analysis showed a significant association between *S.* Give and the presence of a multiphosphoryl transfer protein, the phosphoenolpyruvate-carbohydrate phosphotransferase system (PTS) fructose transporter subunit IIB gene, and PTS fructose transporter subunit IIC genes, which facilitate the uptake of extracellular sugars [87]. In *S.* Give, the sugars transported by PTS might assist in biofilm formation, as reported in *Vibrio cholerae* [88]. Since the mechanism of biofilm formation is complex and regulated by several genes, we speculate that *S.* Give uses both *dam* and *PTS* genes for high biofilm production.

Our research has a few limitations. Our dataset is derived from samples collected over five years in Northeast Ohio only, and similar data from additional states would help to draw more general conclusions. Likewise, the water samples were collected from 2019 to 2021, while soil and manure samples were collected from 2016 to 2021, which might have affected the results of our study. Additionally, information on antibiotic use was insufficient to conduct any association studies on the use of antibiotics and the *Salmonella* prevalence. Our future studies are designed to obtain detailed metadata on management practices, including antibiotic, herbicide, and pesticide (type, frequency, dose, etc.) use. This might provide insights into the presence of AMR genes in *Salmonella* on these farms. Furthermore, data on feed production was not available, making it difficult to rule out the possibility of *Salmonella* contamination in our sample originating from feed [89]. Therefore, further research to assess the presence of *Salmonella* in feed and feed crops such as maize and alfa alfa in these farm settings is needed to understand the contribution of feed and feed crops to the dissemination of *Salmonella* on these farms. The results of such studies may guide the need to conduct larger studies to look at the role of feed and feed crops in the persistence and dissemination of *Salmonella* and its impact on public health, since feed and ingredients for feed formulation are obtained from different sources.

## 4. Materials and Methods

### 4.1. Sample Collection

Soil, poultry manure, dairy manure, pond water, and stream water samples were collected from fourteen SSCFs located in Northeast Ohio from 2016 to 2021 following a previously described protocol [24]. The criteria for selecting the farms were based on their availability for longitudinal sampling throughout the study period. Soil samples were taken from three random sites and mixed in a Nasco Whirl-Pak™ (Fisher Scientific, Waltham, MA, USA). The soil of the farms was amended with either poultry manure (PM) or dairy manure (DM). Dairy heifers housed on a bedded pack during the winter and raised on pasture in the summer were used for the collection of DM. Six to eight fresh manure pats were collected and pooled together in the Whirl-Pak™. Similarly, PM was collected from poultry storage piles at three random sites and pooled. Ten liters of water was collected from either a pond or a stream, based on the source of the water used to irrigate the farm, in a sterile Nalgene gallon. Samples were transferred to the lab in an ice box at 4 °C and processed immediately.

### 4.2. Sample Processing to Isolate Salmonella

Samples were processed following a previously described protocol [24]. Twenty-five grams of (manure and soil) samples were weighed and resuspended in 225 mL of phosphate-buffered saline (PBS), mixed thoroughly to make a slurry, and ten-fold serially diluted. Then, 100 µL of each dilution was plated on a Xylose-Lysine-Tergitol 4 (XLT-4) plate and incubated at 37 °C for 24 h to isolate *Salmonella* directly. Similarly, 100 µL of water was plated on an XLT-4 plate without dilution. In addition, water was concentrated by passing it through a Modified Moore Swab (MMS) filter. The filter was aseptically removed and transferred to Whirl-Pak bags, mixed with 250 mL of PBS, serially diluted, and plated on an XLT-4 plate. Apart from direct plating, 10 mL of each sample was enriched in 90 mL of tetrathionate broth (TTB) for 24 h and plated on an XLT-4 plate as described previously. The isolated black colonies typical of *Salmonella* on XLT-4 plates were stored in BHI broth at −80 °C. 

### 4.3. DNA Extraction

*Salmonella* DNA was extracted using the boiling method [90]. A loopful of *Salmonella* culture was resuspended in 100µL of sterile RNase/DNase-free water and boiled at 95 °C for 10 min. The debris was pelleted by centrifugation at 4000× *g* for 10 min. The supernatant containing the DNA was transferred to new tubes and stored at −20 °C. A total of 2 µL of the lysate was used as a template for PCR for the confirmation of *Salmonella* using the invasion (*invA*) gene (final volume of 25 µL and 35 cycles). The 284 bp amplicon of the *invA* gene was amplified using an initial denaturation at 94 °C for 3 min; denaturation at 94 °C for 1 min, annealing at 53 °C for 2 min, and elongation at 72 °C for 1 min; and a final extension at 72 °C for 7 min [91]. The primers used for PCR were S139F: GTGAAATTATCGCCACGTTCGGGCAA and S141R: TCATCGCACCGTCAAAGGAACC [90]. The size of the PCR product was confirmed by visualizing on a 2% agarose gel, and positive samples were sent for sequencing.

For whole-genome sequencing, DNA was extracted from pure *Salmonella* isolates (*n* = 80) using the Wizard Genomic DNA Purification kit (Promega, Madison, WI, USA) following the manufacturer’s instructions.

### 4.4. Genome Assembly and Annotation

DNA samples were prepared for Illumina MiSeq 2 × 300 bp sequencing using the Nextera DNA Library Preparation Kit (Illumina, South Dakota State University, Brookings, SD, USA). Sequence quality was checked using FastQC v. 0.11.9 [92]. Adapters and poor-quality bases were removed by Trimmomatic v. 0.36 [93] using the quality trimming setting “LEADING: 3 TRAILING: 3 SLIDINGWINDOW:4:20 MINLEN:36”, which removes low-quality bases with a sliding-window approach and subsequently removes reads shorter than 36 bp. Paired-end reads for each isolate were de novo assembled using the SPAdes assembler (v. 3.6.0), specifying k-mer sizes of 21, 33, 55, 77, 99, and 127 [94]. SPAdes uses a de Bruijin graph to assemble reads, in which reads are broken down as k-mers to build the graph. When multiple k-mers are used to make the graphs, multiple graph reduction and correction steps are applied so that the final assembly becomes more complete compared to a single k-mer [95]. Basic assembly statistics, such as the number of contigs, genome size, and GC content, were computed using QUAST v. 5.0.2 [96]. BUSCO v. 5 and CheckM v. 1.2.0 were used to examine the completeness and contamination of the assemblies [97,98,99]. The read coverage was computed by mapping the reads back to the assemblies using BBmap v. 35.49 [100]. Assemblies with >300 contigs and an N50 < 25,000 bp were removed from further analysis [101]. Prokka v. 1.13 [102] was used to annotate each assembly with default settings.

### 4.5. Pan-Genome Construction

Roary v. 3.13 [103] was used to perform a pan-genome analysis in order to examine shared and unique genes among the isolates. The genes were classified into four different classes: core (present in 99–100% of isolates), soft-core (95–99%), shell (15–95%), and cloud (0–15%) genes. Roary uses MAFFT v7. 508 [104] to align nucleotide sequences.

### 4.6. Pan-Genome-Wide Association Studies

Scoary v. 1.6.16 was used to find genes whose presence or absence was significantly associated with each serotype [105]. A gene presence–absence file generated by Roary was used as the genotypic data, and the serotype identity was used as the phenotypic data. Genes with a Benjamini–Hochberg-corrected *p*-value < 0.05 and an odds ratio > 1 were considered significant [106].

### 4.7. Serotypes, Sequence Type (ST), Average Nucleotide Identity (ANI), and Phylogenetic Structure

Serotyping is the gold standard for the classification of Salmonella. SeqSero2 v. 4.6.1 was used for in silico serotyping using the raw sequence reads [107]. SeqSero2 uses a k-mer-based algorithm to detect the serotype [108]. In SeqSero2, the somatic O-antigen (long-chain lipopolysaccharide located on the outer membrane) is detected by the *wzx* flippase gene, the *wzy* polymerase gene, and *rfb* cluster genes. Likewise, the H-antigen (flagellar antigen) is detected by H1 (*fliC*) and H2 (*fljB*) genes [108]. Additionally, the multilocus sequence type (MLST) of each isolate was determined using the command-line tool MLST v. 2.23.0 [109]. To confirm that in silico serotyping was accurate, a phylogenetic tree was constructed using core (i.e., shared) single-nucleotide polymorphisms (SNPs) with the reference-free and alignment-free method implemented in kSNP3 v. 3.1 [110]. This method, which is based on identifying shared short k-mers, was chosen because of its better performance compared to conventional alignment-based [111,112] methods. The kSNP3 K-chooser utility was used to identify the optimal k-mer length, after which kSNP3 was run to identify core SNPs and build a maximum-likelihood phylogeny using Fasttree. The R/Bioconductor package ggtree v. 3.2.1 [113] was used to plot the resulting tree. ANI values were calculated by sourmash v. 4.6.1 to examine the overall genetic similarity among isolates [114].

### 4.8. In Silico Antimicrobial Resistance Determinant (AMR), Virulence, and Plasmids

AMRFinderPlus v. 3.10.30 was used to detect AMR genes and stress response genes [115] within each assembly. The program detects acquired AMR genes as well as resistance-conferring point mutations within genes. The “*Salmonella*” organism option was selected, and default coverage (50%) and identity (90%) thresholds were used [114]. ABRicate v. 1.0.1 was used with the Virulence Factor Database (VFDB; “--db vfdb” option) with minimum identity and coverage thresholds of 70% and 50%, respectively [32], to detect virulence genes, including those from secretion systems, and to identify the *Salmonella* pathogenicity island where the virulence genes were located. Additionally, the MOB-suite v. 3.1.0 [116] utility MOB-typer was used to identify plasmid replicons and predict the mobility of each plasmid. MOB-typer uses three dissemination markers, namely, mobilization protein (relaxase), mating pair formation (MPF) complex, and origin of transfer (*oriT*) genes, to classify the mobility of the plasmid. A plasmid classified as “conjugative” has both relaxase and MPF, “mobilizable” has either relaxase or *oriT* but no MPF, and “non-mobilizable” has neither relaxase nor *oriT* [117,118].

### 4.9. Phenotypic Antimicrobial Susceptibility Testing (AST)

Antimicrobial susceptibility testing (AST) was performed using 16 different antibiotics belonging to 11 different classes. The broth microdilution method was used following the Clinical and Laboratory Standards Institute (CLSI) guidelines [119]. The minimum inhibitory concentration (MIC) of the antibiotics (μg/mL) tested were as follows: streptomycin (STR: 32–64), kanamycin (KAN: 16–64), gentamycin (GEN: 4–16), amoxicillin (AMC: 8–32), ceftriaxone (CRO: 1–4), cefoxitin (CEFO: 8–32), sulfisoxazole (SUL: 256–512), trimethoprim–sulfamethoxazole (TRI: 2–4), azithromycin (AZM: 16–32), meropenem (MEM: 1–4), ampicillin (AMP: 8–32), chloramphenicol (CHL: 8–32), colistin (COL: 2–2), ciprofloxacin (CIP: 0.06–1), nalidixic acid (NAL: 16–32), and tetracycline (TET: 4–16). To determine MIC, *Salmonella* was grown overnight, the optical density (OD) was adjusted to make a final concentration of 5 × 10^6^ CFU/mL, and 10 µL of the culture was added to a well of a 96-well plate containing 90 µL of antibiotics. The plate was incubated at 37 °C for 12 h, and the growth of isolates was visually inspected. The MIC results and CLSI breakpoint were used to classify them as “susceptible”, “intermediate”, or “resistant”. In addition, the intermediate isolates were classified as susceptible isolates to make a correlation plot. Isolates that were resistant to three or more different classes of antibiotics were further classified as multidrug-resistant (MDR) *Salmonella*.

### 4.10. Biofilm Assay

A biofilm assay was performed following a previously described protocol [120]. *Salmonella* was grown overnight and adjusted to an OD of 0.05, and 200 µL was added to 96-well plates to allow for biofilm formation. The plate was incubated at 37 °C for 24 h and washed 3 times to remove non-adherent *Salmonella*. The biofilm was stained with 100 µL crystal violet (0.4% *w*/*v*) for 20 min and washed (3 times) with water, and 100 µL of 33%(*v*/*v*) (*V*/*V*) glacial acetic acid was added to resuspend the biofilm. The OD was measured at 660 nm. The strain was classified according to the amount of biofilm produced based on the following criteria: non-biofilm producer (ODS ≤ ODC), weak biofilm producer (ODC < ODS ≤ 2 × ODC), moderate biofilm producer (2 × ODC < ODS ≤ 4 × ODC), and high biofilm producer (4 × ODC < ODS), where ODC = OD of negative control, and ODS = OD of samples.

### 4.11. Comparative Genomic Analysis of AMR Genes Using Additional Salmonella Genomes

Comparative genomic analysis was performed to check whether our isolates could be potential reservoirs for AMR gene transmission to humans. A total of 2829 *Salmonella* genomes from Ohio were available at NCBI as of 1/31/2023 31 January 2023. Out of those, 51 isolates represented our own samples, and for 77 others, the assembly ID was missing. Therefore, we downloaded a total of 2701 *Salmonella* genome assemblies from NCBI using the NCBI datasets tool (https://github.com/ncbi/datasets (accessed on 31 January 2023). We downloaded the following number of samples for these sample types: 2674 environmental, 23 clinical human, 3 clinical animal, and 1 clinical “missing”. All assemblies were annotated using Prokka v. 1.13, and ARGs were detected using AMRFinderPlus v.3.10.30. Using ARG presence–absence data from these annotations, a PCA was performed using the prcomp function in R to visualize the overall similarity between ARGs in our sample and those from other Ohio samples.

### 4.12. Statistical Analyses

A metric multi-dimensional scale (MDS) plot was constructed in R using the “vegan” package to visualize the geographic distances between farms. The distance matrix was constructed using the geographical distance between farms in miles. The correlation of genotype with phenotype data for antibiotic susceptibility was conducted by converting them to binary variables: phenotypic sensitivity to an antibiotic was represented as 0, and resistance was represented as 1. Similarly, the presence or absence of a specific AMR gene was represented as 1 and 0, respectively. In R, the “cor” function was used to calculate the correlation, “cor.test” was used to measure the significance of the correlation, and the “corrplot” function (from the package corrplot, v. 0.92) was used to visualize the correlation plot. A one-way ANOVA followed by post hoc tests was used to test whether biofilm formation ability differed between serotypes.

## 5. Conclusions

In conclusion, our study identified 15 different serotypes of *Salmonella* circulating on SSCFs in Ohio. Although the prevalence of *Salmonella* is relatively low on SSCFs compared to commercial farms, the presence of MDR genetic determinants, especially the ACSSuT cassette, emphasizes the need for strict biosecurity measures and intervention strategies. Overall, our results highlight the potential risks associated with the transmission of MDR *Salmonella* from SSCFs to humans. This study provides valuable information that can be used to prevent *Salmonella* outbreaks originating from SSCFs and to inform the development of policies aimed at reducing the transmission of MDR *Salmonella* from farm to fork.

## Figures and Tables

**Table 1:** List of Salmonella collected between 2016 and 2021 from four different isolation sources.

Year	Salmonella-Positive Samples	Farm
2016	1	Dairy manure
2017	2	Poultry manure
2017	1	Dairy manure
2017	1	Soil
2018	1	Poultry manure
2018	1	Dairy manure
2019	3	Poultry manure
2019	3	Water
2020	3	Water
2020	3	Dairy manure
2021	10	Water
Total	29	

**Table 2:** Assembly statistics of the *Salmonella enterica* serotypes isolated from SSCFs. For serotypes with more than one isolate, each row represents the average calculated from different isolates per serotype.

Serotype	Coverage	Size (Mb)	Contigs	GC %	BUSCO	N50	Completeness	Contamination	Gene
*S.* Agbeni	35.4	4.5	42	52.2	98.65	382,607	100.0	0.3	4292
*S.* Anatum	66.9	4.6	24	52.2	98.65	562,293	100.0	0.0	4338
*S.* Berta	69.4	4.7	41	52.2	98.65	289,914	100.0	0.0	4465
*S.* Brenderup	76.0	4.7	27	52.2	98.65	497,120	100.0	0.1	4419
*S.* Cerro	69.3	4.6	61	52.2	98.65	174,627	99.9	0.1	4431
*S.* Dublin	55.9	4.9	37	52.1	98.65	406,024	99.7	0.3	4824
*S.* Enteritidis	64.9	4.6	32.5	52.1	98.65	440,347	100.0	0.2	4444
*S.* Give	78.7	4.5	29	52.2	98.65	454,909	100.0	0.0	4378
*S.* Hartford	36.8	4.8	60	52.1	98.65	236,095	99.4	0.1	4592
*S.* Litchfield	76.3	4.4	52	52.2	98.65	252,267	100.0	0.0	4391
*S.* Montevideo	71.3	4.6	31.8	52.3	98.65	433,986	100.0	0.2	4348
*S.* Muenchen	78.2	4.6	29	52.2	98.65	521,499	100.0	0.0	4345
*S.* Newport	62.7	4.8	36	52.1	98.65	563,829	100.0	0.1	4691
*S.* Oranienburg	53.2	4.6	33	52.1	98.65	542,191	100.0	0.0	4335
*S.* Paratyphi	82.4	4.6	29	52.3	98.65	369,141	100.0	0.5	4309

**Table 3:** Serotype, serogroup, and sequence type classification of *Salmonella* assemblies.

Serotype	Number	Serogroup	MLST
*S.* Anatum	2	E1	64
*S.* Agbeni	1	G	2606
*S.* Braenderup	5	C1	22
*S.* Berta	2	D1	435
*S.* Cerro	23	K	367, 9243
*S.* Dublin	1	D1	10
*S.* Enteritidis	2	D1	11
*S.* Give	6	E1	654
*S.* Hartford	7	C1	405
*S.* Litchfield	1	C2–C3	214
*S.* Montevideo	5	C1	4, 138
*S.* Muenchen	3	C2–C3	83
*S.* Oranienburg	10	C1	23
*S.* Newport	9	C2–C3	45, 350
*S.* Paratyphi B var. L(+) tartrate+	1	B	3927

**Table 4:** Seven antimicrobial-resistant genes detected in 12 samples and 5 serotypes.

Sample_Id	Serotypes	Gene	Antibiotic Class
AG21-0051	Dublin	*blaCMY-2*, *sul2*, *aph(3″)-Ib*, *aph(6)-Id*, *tet(A)*, *floR*	Cephalosporin, sulfonamide, streptomycin, streptomycin, tetracycline, chloramphenicol/florfenicol
AG21-0060	Enteritidis	*gyrA_D87Y*	Quinolone
AG21-0066	Enteritidis	*gyrA_D87Y*	Quinolone
AG21-0012	Newport	*floR*, *tet(A)*, *aph(6)-Id*, *aph(3″)-Ib*, *sul2*, *blaCMY-2*	Chloramphenicol/florfenicol, tetracycline, streptomycin, streptomycin, sulfonamide, cephalosporin
AG21-0014	Newport	*floR*, *tet(A)*, *aph(6)-Id*, *aph(3″)-Ib*, *sul2*, *blaCMY-2*	Chloramphenicol/florfenicol, tetracycline, streptomycin, streptomycin, sulfonamide, cephalosporin
AG21-0584	Newport	*floR*, *tet(A)*, *aph(6)-Id*, *aph(3″)-Ib*, *sul2*, *blaCMY-2*	Chloramphenicol/florfenicol, tetracycline, streptomycin, streptomycin, sulfonamide, cephalosporin
AG21-0586	Newport	*floR*, *tet(A)*, *aph(6)-Id*, *aph(3″)-Ib*, *sul2*, *blaCMY-2*	Chloramphenicol/florfenicol, tetracycline, streptomycin, streptomycin, sulfonamide, cephalosporin
AG21-0587	Newport	*floR*, *tet(A)*, *aph(6)-Id*, *aph(3″)-Ib*, *sul2*, *blaCMY-2*	Chloramphenicol/florfenicol, tetracycline, streptomycin, streptomycin, sulfonamide, cephalosporin
AG21-0050	Montevideo	*fosA7*	Fosfomycin
AG21-0589	Muenchen	*aph(3″)-Ib*, *sul2*, *tet(A)*	Streptomycin, sulfonamide, tetracycline
AG21-0590	Muenchen	*aph(3″)-Ib*, *sul2*, *tet(A)*	Streptomycin, sulfonamide, tetracycline
AG21-0591	Muenchen	*aph(3″)-Ib*, *sul2*, *tet(A)*	Streptomycin, sulfonamide, tetracycline

## Data Availability

The DNA sequencing reads from this study were deposited into the NCBI SRA: SRP083283 under PRJNA338674 and PRJNA280335.

## Supplementary Materials

The following supporting information can be downloaded at https://www.mdpi.com/article/10.3390/antibiotics12111637/s1: Figure S1: Functional annotation of the core (A) and accessory (B) genes using the cluster of orthologous genes (COG); Figure S2: Multi-dimensional scaling (MDS) plot constructed using the geographical location of the farms; Table S1: The frequency of samples collected from each farm; Table S2: The genome statistics of 78 isolates; Table S3: The genes significantly associated with each serotype by Scoary; Table S4: List of all detected plasmids; Table S5: List of all detected virulence genes.
