# Supplementary material for: Genomic Diversity, Antimicrobial Resistance, Plasmidome, and Virulence Profiles of Salmonella Isolated from Small Specialty Crop Farms Revealed by Whole-Genome Sequencing
Source: Antibiotics (Basel). 2023 Nov 18;12(11):1637. doi: 10.3390/antibiotics12111637 (PMC10668983; doi:10.3390/antibiotics12111637)
Supplement: Supplementary file 1 [file antibiotics-12-01637-s001.zip › supplementary Table S1.number of samples-collected from each farm 11 10 2023.pdf]

Table S1. The total number of samples and frequency of samples collected from each farm.

Farm	Total Samples Collected	Soil	Manure	Water
A	94	55 (once a month)	39 (once a month)	not collected
B	89	27 (once a month)	58 (once - twice a month)	4 (once a month)
C	159	77 (twice a month)	64 (twice a month)	18 (once a month)
D	102	39 (once a month)	63 (once - twice a month)	not collected
E	26	26 (once a month)	not collected	not collected
F	118	45 (once a month)	37 (once a month)	36 (once a month)
G	144	61 (once - thrice a month)	47 (once - twice a month)	39 (once - twice a month)
H	102	50 (once - twice a month)	38 (once - twice a month)	15 (once a month)
I	74	67 (once - twice a month)		7 (once a month)
J	145	62 (once - thrice a month)	47 (twice a month)	36 (once - twice a month)
K	55	63 (once - twice a month)	20 (once every 2 months)	16 (once - twice a month)
L	136	62 (once - twice a month)	37(once - twice a month)	24 (once - twice a month)
O	7	not collected	7 (once a month)	not collected
P	1	1 (1 time)	not collected	not collected
Total	1252			

Dairy manure, poultry manure and soil samples were collected from 2016-2021, whereas water was collected from 2019-2021.The frequency of samples collected from each farm is provided in the parentheses.
