# Supplementary material for: Genomic Diversity, Antimicrobial Resistance, Plasmidome, and Virulence Profiles of Salmonella Isolated from Small Specialty Crop Farms Revealed by Whole-Genome Sequencing
Source: Antibiotics (Basel). 2023 Nov 18;12(11):1637. doi: 10.3390/antibiotics12111637 (PMC10668983; doi:10.3390/antibiotics12111637)
Supplement: Supplementary file 1 [file antibiotics-12-01637-s001.zip › Supplementary_file_figures.pdf]

Supplementary file

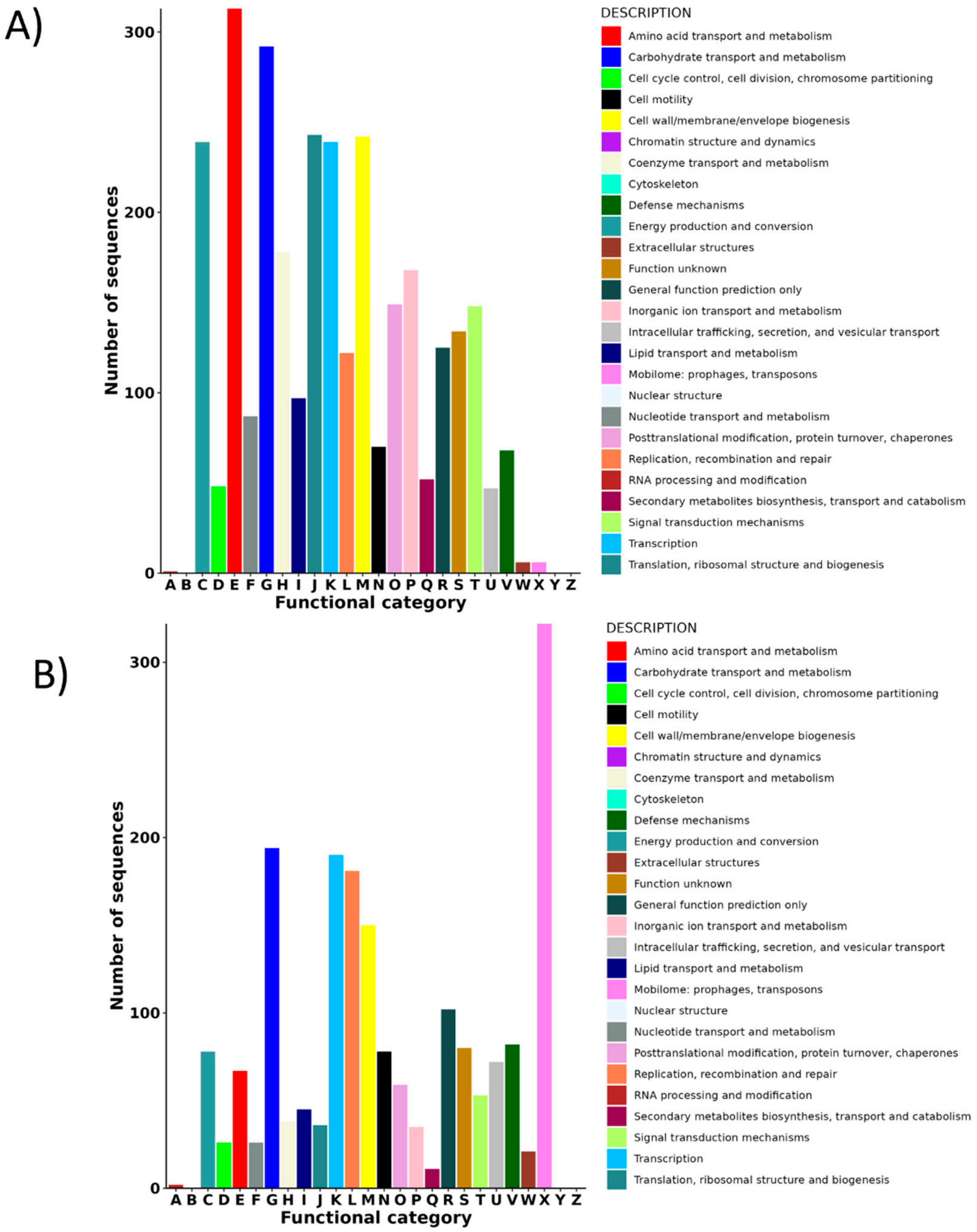

Figure S1. Functional annotation of the core(A) and the accessory(B) genes using the cluster of orthologous genes (COG).

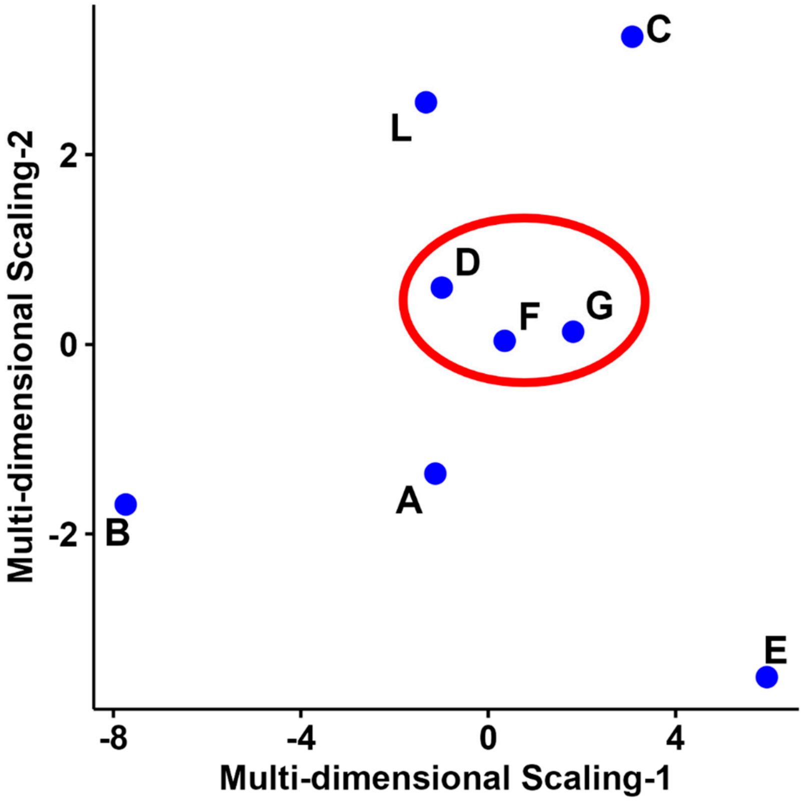

Figure S2. Multi-dimensional scaling (MDS) plot constructed using the geographical location of the farms (8) in R. The distance matrix was computed using each farm's latitude and longitude coordinates. Out of 8 farms, 3 Farms (D, F, and G) were located closer to each other.
